# Utilizing Macrophages Missile for Sulfate-Based Nanomedicine Delivery in Lung Cancer Therapy

**DOI:** 10.34133/research.0448

**Published:** 2024-08-14

**Authors:** Chang Liu, Yongyang Chen, Xiaoyu Xu, Miao Yin, Hongbo Zhang, Wenmei Su

**Affiliations:** ^1^ Department of Pulmonary Oncology, Affiliated Hospital of Guangdong Medical University, Zhanjiang, 524001, China.; ^2^ Guangdong Provincial Key Laboratory of Autophagy and Major Chronic Non-communicable Diseases, Affiliated Hospital of Guangdong Medical University, Zhanjiang, 524001, China.; ^3^ Pharmaceutical Sciences Laboratory, Faculty of Science and Engineering, Åbo Akademi University, Turku, 20520, Finland.; ^4^ Turku Bioscience Centre, University of Turku and Åbo Akademi University, Turku, 20520, Finland.; ^5^ ENT Institute and Department of Otorhinolaryngology, Eye & ENT Hospital, State Key Laboratory of Medical Neurobiology, Institutes of Biomedical Sciences, Fudan University, Shanghai, 200031, China.

## Abstract

Nanomaterial-based drug delivery systems are susceptible to premature drug leakage and systemic toxicity due to lack of specific targeting, and live-cell drug delivery is also prone to be restricted by drug carrier–cell interactions. Here, a method is established to adsorb drug-loaded nanomaterials externally to the live cells, which reduces cytotoxicity caused by drug uptake and improves the bioactivity of the carrier cells and drug release at the lesion site. It was found that polyphenols act like “double-sided tape” to bridge metal–organic framework (MOF) nanoparticles with live macrophages (Mφ), attaching MOFs to the Mφ surface and minimizing intracellular uptake, with no negative effect on cell proliferation. On this basis, a “macrophage missile” with peroxymonosulfate (PMS)-loaded MOF nanoparticles on the cell surface was constructed. As a “propellant”, the Mφ, in which bioactivity is preserved, can selectively identify and target tumor cells, precisely bringing nanomedicines to the lesion. MOF nanoparticles are used to load and catalyze PMS, which acts as an exogenous source of reactive oxygen species, showing higher efficacy and lower toxicity in an oxygen-independent manner. The primary study results demonstrate that this innovative combination of biology and nanomaterials remarkably enhances tumor targeting and therapeutic efficacy while reducing systemic side effects. This approach is expected to provide a more effective and safer treatment for lung cancer and holds promise for broader applications in other cancer therapies.

## Introduction

Lung cancer remains one of the most common and highly aggressive malignant tumor characterized by its early occult nature, rapid metastasis, and poor prognosis [1]. Although there are advancements in treatments such as surgery, radiotherapy, chemotherapy, targeted therapies and immunotherapy [2–6], the considerable challenges are still present. These include limitations related to the adaptability of surgery to tumor location and size, drug resistance, and potential adverse effects, all of which constrain the effectiveness and survival benefits of patients with lung cancer. Therefore, there is a critical need to develop innovative treatment strategies that can address these challenges more effectively. Recent advances in stimuli-responsive tumor therapies, such as photodynamic therapy (PDT), photothermal therapy (PTT), and chemical dynamic therapy (CDT) [7–9], have attracted considerable attention due to their precision and controllability. These treatment methods utilize external or internal stimuli to activate these nanodrugs, increasing reactive oxygen species (ROS) levels to induce cellular effects such as apoptosis and necrosis, thereby enhancing treatment efficacy and reducing systemic toxicity [10]. However, the unique characteristics of the tumor microenvironment, including hypoxia, insufficient endogenous H_2_O_2_ levels to support ROS production, and rapid ROS clearance, limit the effectiveness and broader application of stimuli-responsive nanotherapy [11–13]. Sulfate radicals (·SO_4_^−^) have recently emerged as a potential solution to these challenges due to their longer half-life, faster reaction rate, and stronger selectivity, making them a more potent form of ROS [14]. These radicals can be generated independently of H_2_O_2_ or O_2_ participation within a pH range of 2 to 9 by activating persulfates (S_2_O_8_^−^) and peroxymonosulfates (PMSs: HSO_5_^−^) [15,16]. Specifically, PMS is considered easier to activate due to its asymmetric structure. For instance, phospholipid-coated Na_2_S_2_O_8_ nanoparticles can produce ROS (·SO_4_^−^ and ·OH) without being influenced by H_2_O_2_, O_2_, or pH values [17]. Despite their potential, the application of sulfate radicals in cancer nanotherapy is still relatively unexplored, necessitating further research into their mechanisms and prospects in treatment.

In developing an effective PMS-based anticancer system, metal–organic frameworks (MOFs) have attracted widespread interest due to their unique structure and properties. MOFs, assembled from metal ions/clusters and organic linkers, exhibit high porosity, tunable pore size, and high surface area, making them suitable for various applications, including drug delivery and nanozyme for cancer therapy. They can be functionalized with targeting molecules for active delivery purposes, including antibodies, peptides, aptamers, small molecules, and polysaccharides. However, some modified MOFs are unstable in physiological conditions, which may cause serious issues, such as decomposition of targeting moieties, short circulation time, and premature drug release, limiting their biomedical application noticeably. The live-cell-based drug delivery systems present a promising option to overcome some of the shortcomings of nanocarrier delivery in treating various diseases [18–20]. For example, immune cells such as macrophages (Mφ) [21–23], neutrophils, T cells, and natural killer cells can respond to signaling molecules at the disease site, enabling targeted drug delivery and therapy [24–26]. However, live-cell-based drug delivery faces challenges such as how to maintain cell functions while carrying drugs and is largely limited by adverse drug carrier–cell interactions. Premature drug leakage into the cytoplasm can impair the biological activity of carrier cells, while drug-loaded cells may have difficulty releasing drugs or cause drug inactivation in cellular environments such as lysosomes, leading to reduced efficacy. Adhesion of drug-carrying nanoparticles to cell surfaces, by comparison, presents several advantages, including minimal cellular damage and easier drug distribution at the site of diseases. Metal polyphenolic networks, formed by the coordination of metal ions from MOFs with polyphenolic ligands have adhesive properties from polyphenolic molecules [27,28]. This characteristic allows drug-loaded MOF nanoparticles to attach to the surface of living cells, minimizing their endocytosis, prolonging the biological activity of carrier cells, and facilitating drug release at disease sites, thereby enhancing treatment efficacy.

**Fig. 1.**
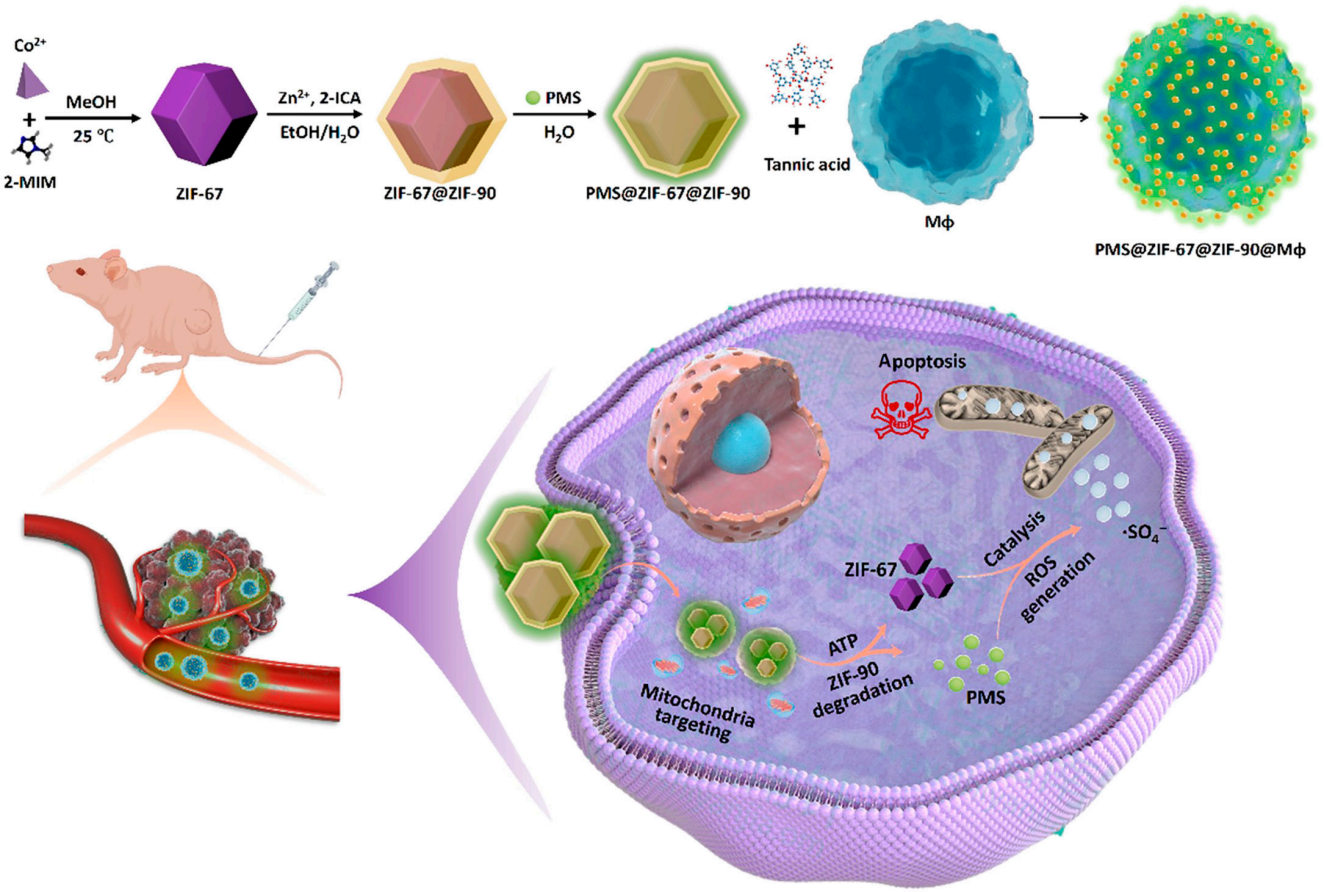
Schematic illustration for the fabrication and theragnostic functions of “macrophages missile” used for sulfate radical nanotherapy of lung cancer.

This study introduces the concept of “macrophages missile”, inspired by polyphenol bridging MOF and Mφ (Fig. 1). The PMS-loaded MOFs bind to Mφ through metal–polyphenol coordination and a variety of hydrogen bonding interactions at the polyphenol–cell interface, maintaining the biological activity of the carrier cells and enhancing the targeting and catalytic therapeutic efficacy against tumor cells. Through mitochondrial targeting, the release of zeolitic imidazolate framework-67 (ZIF-67) under adenosine triphosphate (ATP) degradation catalyzes PMS to produce ·SO_4_^−^, inducing mitochondrial apoptosis and leading to tumor cell death. This facile, benign, and efficient live-cell-based drug delivery system demonstrates important potential for clinical translation in cancer therapy. Optimized preparation of PMS@MOF@Mφ has proven superior performance both in vitro and in vivo. Overall, this concept combines the unique properties of MOFs with the biological function of Mφ, providing an innovative and effective platform for tumor treatment.

## Results

### Synthesis and characterization of core–shell MOF for enhanced PMS activation

**Fig. 2.**
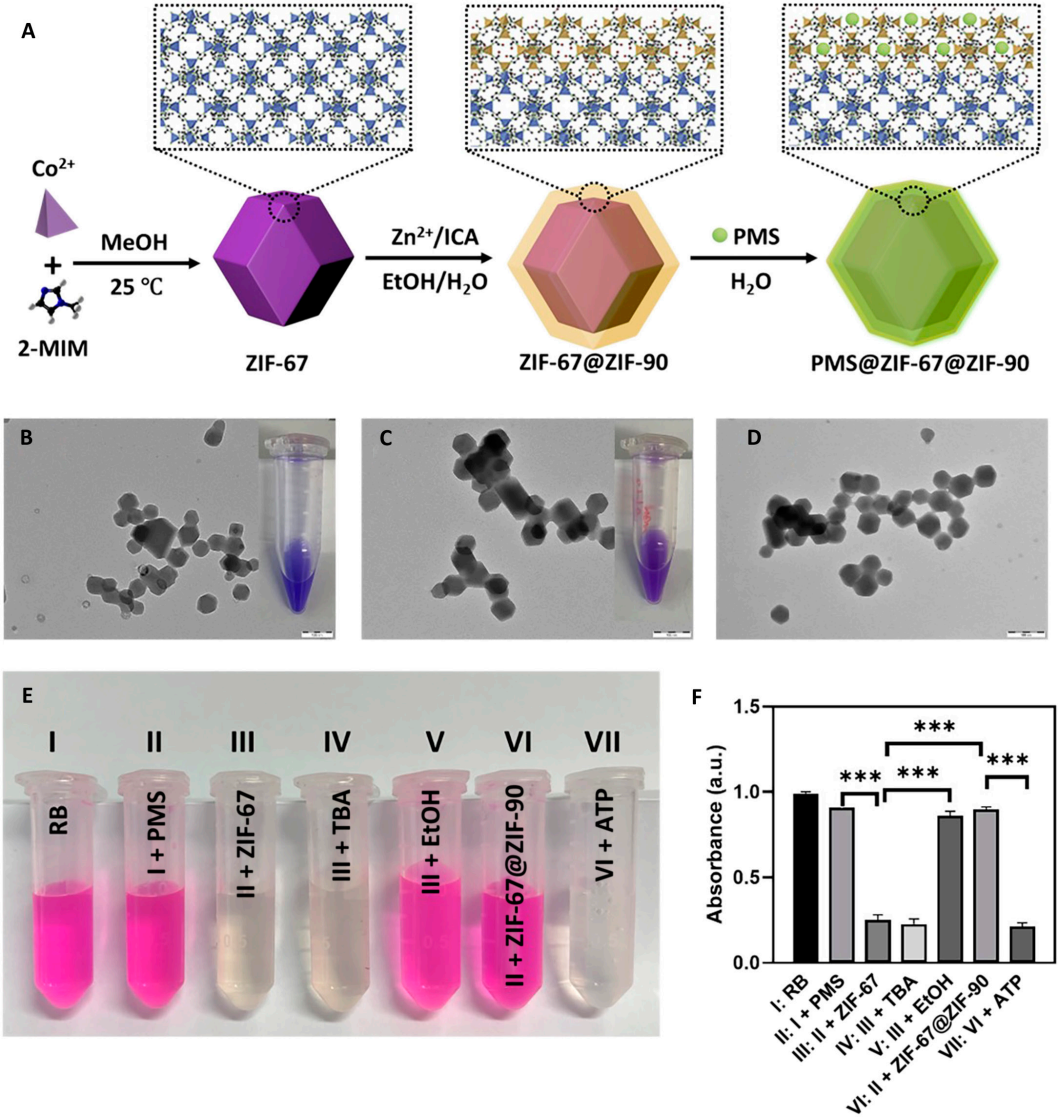
Synthesis, characterization, and catalytic performance of core–shell ZIF-67@ZIF-90. (A) Schematic illustration of the preparation of core–shell ZIF-67@ZIF-90 and PMS@ZIF-67@ZIF-90. TEM images and digital photographs of ZIF-67 (B), ZIF-67@ZIF-90 (C), and PMS@ZIF-67@ZIF-90 nanoparticles (D). (E and F) Degradation of RB due to ∙SO_4_^−^ generation: (I) RB as control, (II) tube I with 150 μg·ml^−1^ PMS, (III) tube II with 10 μg·ml^−1^ ZIF-67, (IV) tube III with quencher tetra-butanol (TBA), (V) tube III with quencher ethanol, (VI) tube II with 10 μg·ml^−1^ ZIF-67@ZIF-90, and (VII) tube VI with 1.0 mM ATP. Data were shown as mean ± standard error of the mean. *P < 0.05, **P < 0.01, ***P < 0.001, ****P < 0.0001.

The nanosized ZIF-67 particles were fabricated according to previously reported methods [29]. Using these ZIF-67 nanoparticles as both initial and core materials, the MOF core–shell structure was constructed step by step (Fig. 2A). Transmission electron microscopy (TEM) images displayed that the as-prepared ZIF-67 was uniformly shaped in a well-defined rhombic dodecahedron with an average size of about 50 ± 10 nm (Fig. 2B). This was consistent with the dynamic light scattering (DLS) hydrodynamic diameter distribution curves shown in Fig. S1A and scanning electron microscopy image as shown in Fig. S2A. The core–shell MOFs (ZIF-67@ZIF-90) were fabricated by a seed-mediated epitaxial growth method. As shown in Fig. 2C, there was only a slight increase in their morphological parameters, including particle diameter, same as the DLS size in Fig. S1A and scanning electron microscopy image in Fig. S2B. After coating the ZIF-90 shell, which has an intrinsic light-yellow color, the formation of the ZIF-67@ZIF-90 can be revealed by the distinct change in color from dark blue to purple shown in the inserted images of Fig. 2B and C. Compared with pure ZIF-67 or ZIF-90 nanoparticles, the core–shell MOF nanoparticles exhibited 2 main absorption peaks at 204 and 293 nm attributed to the corresponding organic ligands, indicating the coating of the ZIF-90 shell around the ZIF-67 core (Fig. S3) [30]. The presence of the ZIF-90 shell is further evidenced by a new distinctive peak at 1668 cm^−1^ in the C=O band from imidazole-2-carboxaldehyde (2-ICA) (Fig. S4). The zeta potential analyses were employed to determine the surface charge density of the dispersed nanoparticles (Fig. S1B), showing measured zeta potential values of 2.88 and 4.85 mV for ZIF-67 and ZIF-67@ZIF-90, respectively, with a more positive change in surface charge due to the electron-withdrawing functional groups (–COH) on the ZIF-90 shell. The increase in zeta potential confirms the successful coating of ZIF-90 around the ZIF-67 core, enhancing the stability and functional properties of the nanoparticles. Additionally, similar TEM observations were made for the PMS-loaded nanoparticles (PMS@ZIF-67@ZIF-90, Fig. 2D). To investigate whether ZIF-67 can activate PMS to produce ·SO_4_^−^, we incubated PMS with ZIF-67 in the presence of Rhodamine B (RB), a dye indicator that can be oxidized by oxidative radicals to the colorless. The RB aqueous solution remained pink when it was incubated with PMS (Tube II) as shown in Fig. 2E, which indicates that PMS has very limited oxidative capability in the absence of an activator. In contrast, the RB aqueous solution turned colorless immediately upon cotreatment with PMS and ZIF-67 (Tube III), showing the strong oxidative capability of PMS catalyzed by cobalt ions in ZIF-67. Since ·SO_4_^−^ and ∙OH were both present in this solution, a quenching assay was conducted to identify which radical was the primary oxidizing agent. Tert-butyl alcohol (TBA) has been reported to quench ∙OH, whereas ethanol tapically reacts with ∙SO_4_^−^ and ∙OH simultaneously [31,32]. The oxidation of RB by PMS with ZIF-67 was absolutely affected by ethanol (Tube V) rather than TBA (Tube IV) shown in Fig. 2E, which indicates that superior oxidative performance of PMS with ZIF-67 system is largely ascribed to ·SO_4_^−^. Furthermore, the preformed ZIF-67@ZIF-90 was used to test the shielding effect of ZIF-90 on the kernel ZIF-67 for catalyzing PMS. The results showed that there is no obvious color change of RB (Tube VI). In addition, ZIF-90 responded to ATP, suggesting that ATP initiated the decomposition of the shell, and in the subsequent catalytic experiments, it was also observed that RB became colorless (Tube VII), which means the ZIF-90 shell can protect the catalytic core from potential deactivation and provide a controlled drug release. The combination of ZIF-67 and ZIF-90 in a core–shell structure leverages their unique chemical properties to create an effective system for catalysis and drug delivery, demonstrating outstanding potential for cancer therapy.

### Construction and effects of surface-attached nanoparticles on Mφ

**Fig. 3.**
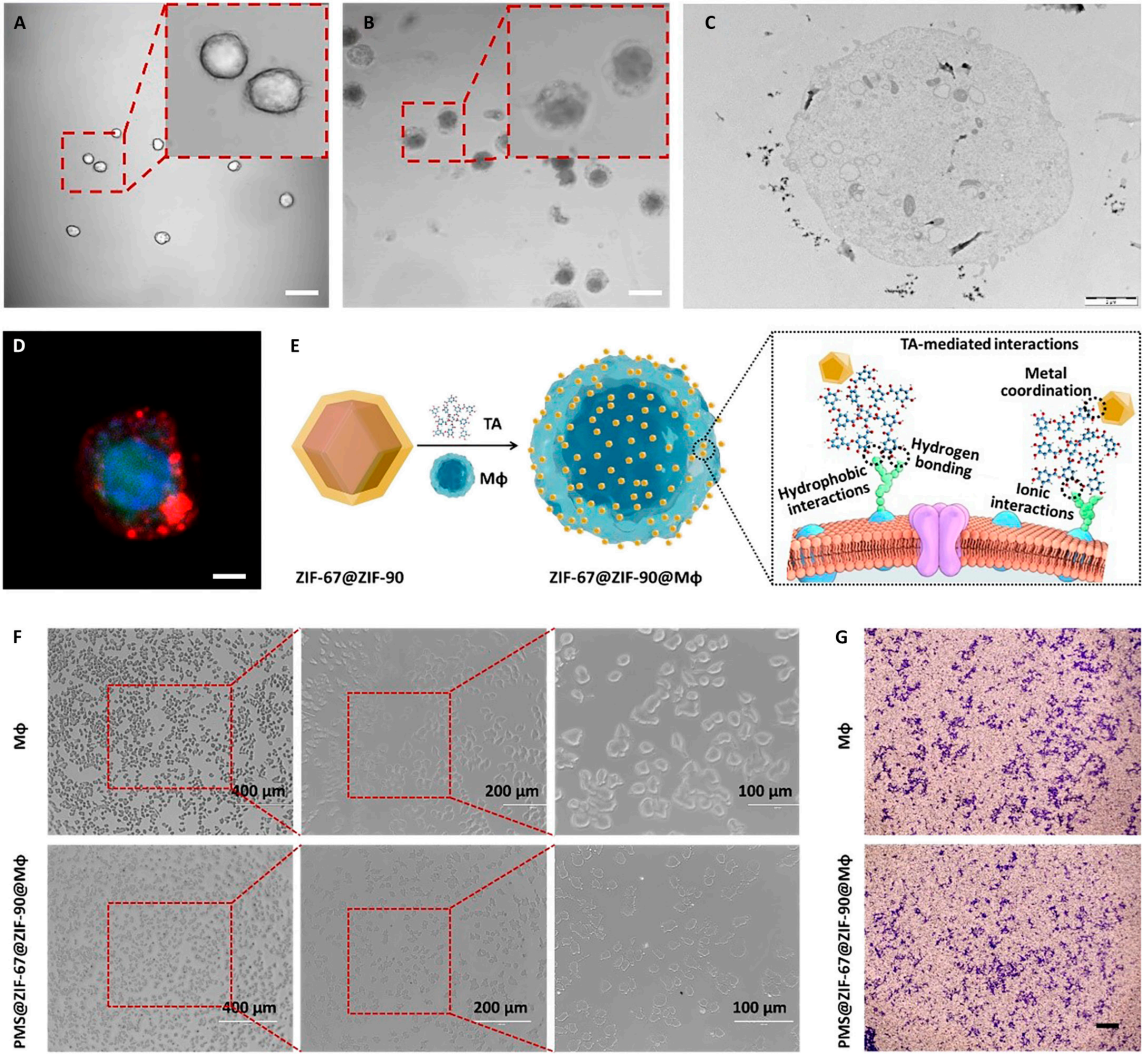
Characterization and functional analysis of ZIF-67@ZIF-90@Mφ for drug delivery applications. Bright-field images of nude Mφ (A) and ZIF-67@ZIF-90@Mφ (B). (C) TEM image of the sectioned ZIF-67@ZIF-90@Mφ nanoparticles. (D) Z-stack CLSM image of ZIF-67@ZIF-90@Mφ demonstrating the formation of ZIF-67@ZIF-90 out layer on Mφ surface (red fluorescence from Cy5.5-labeled ZIF-67@ZIF-90 nanoparticles). (E) Schematic illustration of the fabrication of Mφ with surface-attached MOF nanoparticles for drug delivery and the proposed polyphenol-mediated interactions between metal-coordinated nanoparticles and Mφ surface. (F) Effect of PMS-loaded surface-adsorbed ZIF-67@ZIF-90 nanoparticles on Mφ proliferation. (G) Effect of PMS loading on Mφ migration toward conditioned LLC culture medium.

As a demonstration of the macrophages missile concept, individual murine Mφ (RAW264.7) with surface-adsorbed ZIF-67@ZIF-90 nanoparticles, termed ZIF-67@ZIF-90@Mφ, were constructed through a sequential addition method of a colloidal MOF solution and tannic acid (TA) to cell suspensions prepared in phosphate-buffered saline (PBS) solution. Owing to the robust multivalent metal–phenolic coordination, only a 1-min incubation was required to terminate the intracellular endocytosis of the MOF nanostructured modules through TA-mediated interparticle binding [33]. The MOFs-attached Mφ were directly observed using bright-field imaging of both normal Mφ and MOF@Mφ (Fig. 3A and B). Compared to smooth, naked Mφ, the MOF@Mφ on bright-field images provides the convincing evidence that all individual Mφ had a distinct conformal out layer. TEM images visually confirmed that the MOF nanoparticle were adhered to Mφ surface (Fig. 3C and Fig. S5). Notably, the cell surface was only partially coated by the attached MOFs, which assured the retention of cellular bioactivity. The heterogeneous distribution of the MOF nanoparticles on the Mφ surface may be due to the insufficient mixing of Mφ and ZIF-67@ZIF-90 nanoparticles. Confocal laser scanning microscope (CLSM) images showed that the red fluorescent Cy5.5-labeled ZIF-67@ZIF-90 was attracted on the Mφ surface by TA-assisted adhesion (Fig. 3D), where coherent, conformal MOF nanoparticles anchoring on the Mφ were observed. A schematic of the interactions between the MOF nanoparticles and the Mφ surface was presented in Fig. 3E. These effects consist of the metal polyphenol coordination, the TA-mediated ionic interactions, hydrogen bonding, and hydrophobic interactions with the amino acids of the membrane proteins [34,35]. These interactions are crucial for the stable adsorption of MOF nanoparticles on the cell carrier, providing a practical basis for the successful assembly of the macrophages missile.

Next, the impact of surface attachment of MOF nanoparticles on the bioactivity of the carrier cells and whether it keeps a higher and prolonged activity was further explored. The real-time monitoring of the effect of administered surface adhesion for drug delivery on Mφ viability and proliferation was performed (Fig. 3F). The PMS-loaded MOF nanoparticles attached on cells is well proportional to the added concentrations. The observations made within 48 h before cell confluency revealed that PMS@ZIF-67@ZIF-90@Mφ exhibited an overlapping growth profile with single Mφ at the same initial cell concentrations, indicating good biocompatibility and nontoxicity of both ZIF-67@ZIF-90 nanoparticles and PMS for the carrier cells. Furthermore, the cell migration assay was conducted to evaluate whether PMS-loaded macrophage missile still have their tumor targeting capability. Mφ with nanoparticles adhering to the surface also formed pseudopods, which are essential for their locomotion (Fig. 3G) [36]. It was observed that cells with surface-attached PMS@ZIF-67@ZIF-90 transmigrated more efficiently in response to the chemotaxis of Lewis lung carcinoma (LLC) conditioned medium, compared to single Mφ. This observation aligns with the better preservation of Mφ viability provided by surface adherence. The assembly did not change the Mφ-specific phenotypes, which avoids the possible adverse effects of tumor promotion. These findings further support the feasibility and effectiveness of MOF nanoparticles as potential drug carriers for cancer therapy.

### Tumor-specific uptake, intracellular escape, and mitochondria targeting by macrophages missile

**Fig. 4.**
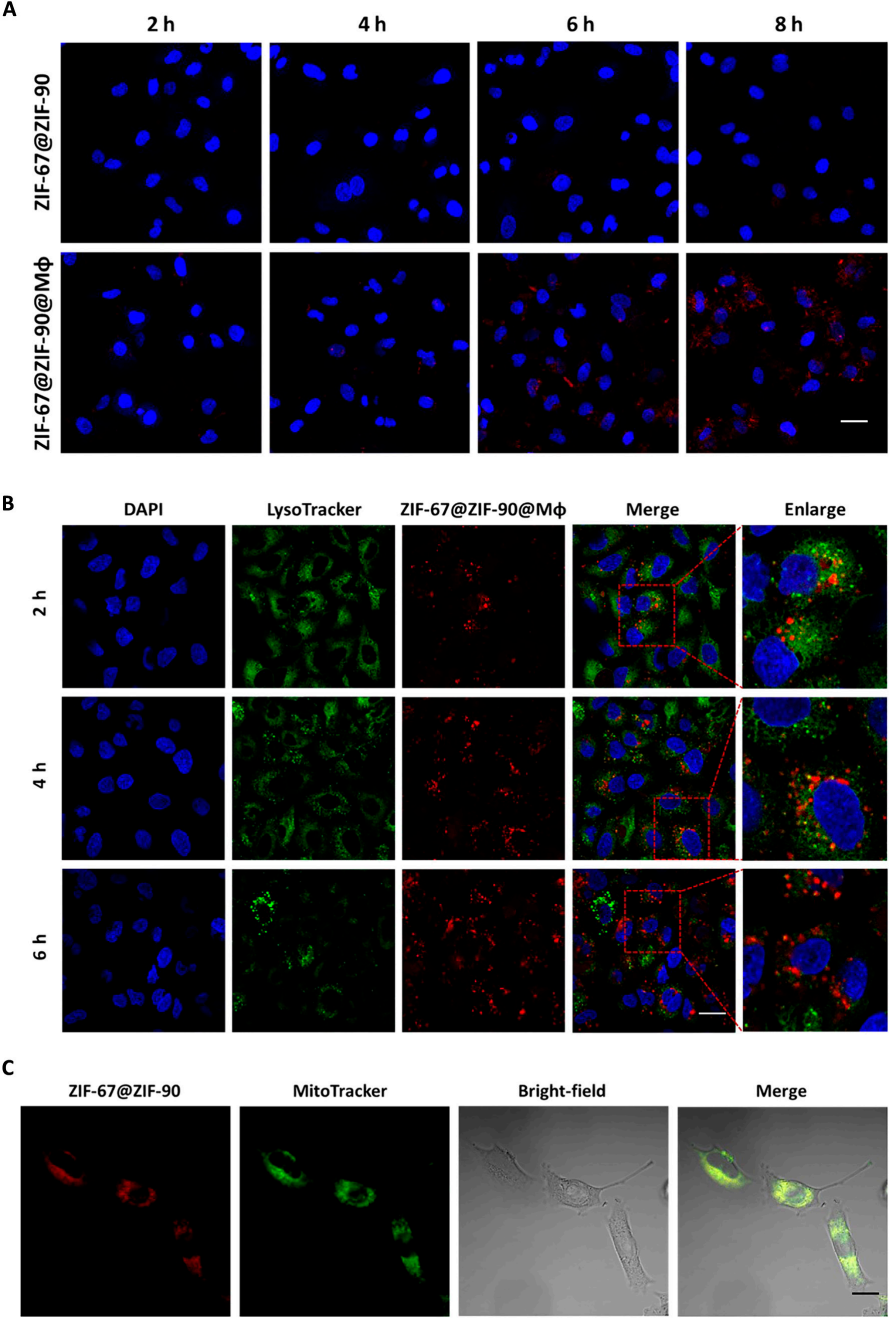
Targeted endocytosis, endosomal escape, and mitochondria targeting analysis of the nanomaterials in cancer cells. (A) CLSM images of LLC cells after treatment with ZIF-67@ZIF-90 and ZIF-@ZIF-90@Mφ for different time intervals (2, 4, 6, and 8 h; scale bar = 20 μm). (B) CLSM images of LLC cells cultured with ZIF-@ZIF-90@Mφ for different time intervals (2, 4, and 6 h). The red fluorescence represents Cy5.5-labeled nanoparticles; the green fluorescence represents lysosomes labeled with LysoTracker@Green staining, and the nuclei are stained blue with 4′,6-diamidino-2-phenylindole (DAPI) (scale bar = 20 μm). (C) CLSM images of LLC cells after 6 h of incubation with ZIF-67@ZIF-90@Mφ (100 μg·ml^−1^) and 15 min of incubation with MitoTracker. The red fluorescence represents Cy5.5-labeled nanoparticles; the green fluorescence represents mitochondria labeled with MitoTracker@Green staining (scale bar = 10 μm).

To evaluate the tumor specificity of the Mφ vector, we labeled ZIF-67@ZIF-90 nanoparticles with red fluorescent Cy5.5, and their uptake by LLC cells was detected. The results showed that the endocytosis of ZIF-67@ZIF-90@Mφ by LLC was stronger compared with that of pristine ZIF-67@ZIF-90, and the fluorescence intensity was dramatically higher in the ZIF-67@ZIF-90@Mφ group (Fig. 4A). Quantitative flow cytometry analysis provides further support that the fluorescence increased over time in ZIF-67@ZIF-90@Mφ-treated LLC cells compared to ZIF-67@ZIF-90 (Fig. S6A). To be therapeutically effective, the ZIF-67@ZIF-90 is required to engage with certain intracellular ingredients, which demands that it escape from the lysosome to avoid being degraded and cleared. The CLSM images of LLC cells treated with ZIF-67@ZIF-90@Mφ under the targeting effect of the Mφ, illustrated in Fig. 4B, showed that the green fluorescence LysoTracker-labeled lysosomes almost overlapped with the red fluorescence representing ZIF-67@ZIF-90 within 4 h and displayed a split between green and red fluorescence at 6 h, suggesting that the nanoparticles successfully escaped from the lysosomes. The colocalizations analysis of ZIF-67@ZIF-90@Mφ and lysosome by Pearson’s correlation coefficient showed that ZIF-67@ZIF-90 entered lysosomes the most at 4 h (Fig. S6B). When ZIF-67@ZIF-90@Mφ nanoparticles were incubated with cells for 6 h, ZIF-67@ZIF-90 began to escape from lysosomes. These findings indicate that effective lysosomal escape is critical for the nanoparticles to fulfil their therapeutic purposes [37].

High tumor-targeting specificity and efficient endocytosis of ZIF-67@ZIF-90@Mφ delivery system has well progresses its use in mitochondria targeting in live cells, as evidenced by culturing LLC cells with Cy5.5-labeled ZIF-67@ZIF-90@Mφ and performing the cellular observation by CLSM. For example, ZIF-67@ZIF-90@Mφ-treated cells had an intense intracellular fluorescence signal, illustrated in Fig. 4C, demonstrating that detached ZIF-67@ZIF-90 nanoparticles have good cell membrane permeability for mitochondrial targeting. To further investigate the organelle localization of dissociated ZIF-67@ZIF-90, the ZIF-67@ZIF-90@Mφ-treated LLC cells were stained with MitoTracker@Green and imaged by CLSM. There was a visible colocalization of ZIF-67@ZIF-90 with MitoTracker@Green, further confirming the preferential accumulation of ZIF-67@ZIF-90 nanoparticles in mitochondria. This may be due to the electrostatic interaction between positively charged ZIF-90 and negatively charged inner membrane mitochondria, which facilitates the preferential aggregation of ZIF-67@ZIF-90 within the mitochondria [38].

### Enhanced antitumor effects of PMS@ZIF-67@ZIF-90@Mφ via mitochondrial targetingand ROS production

**Fig. 5.**
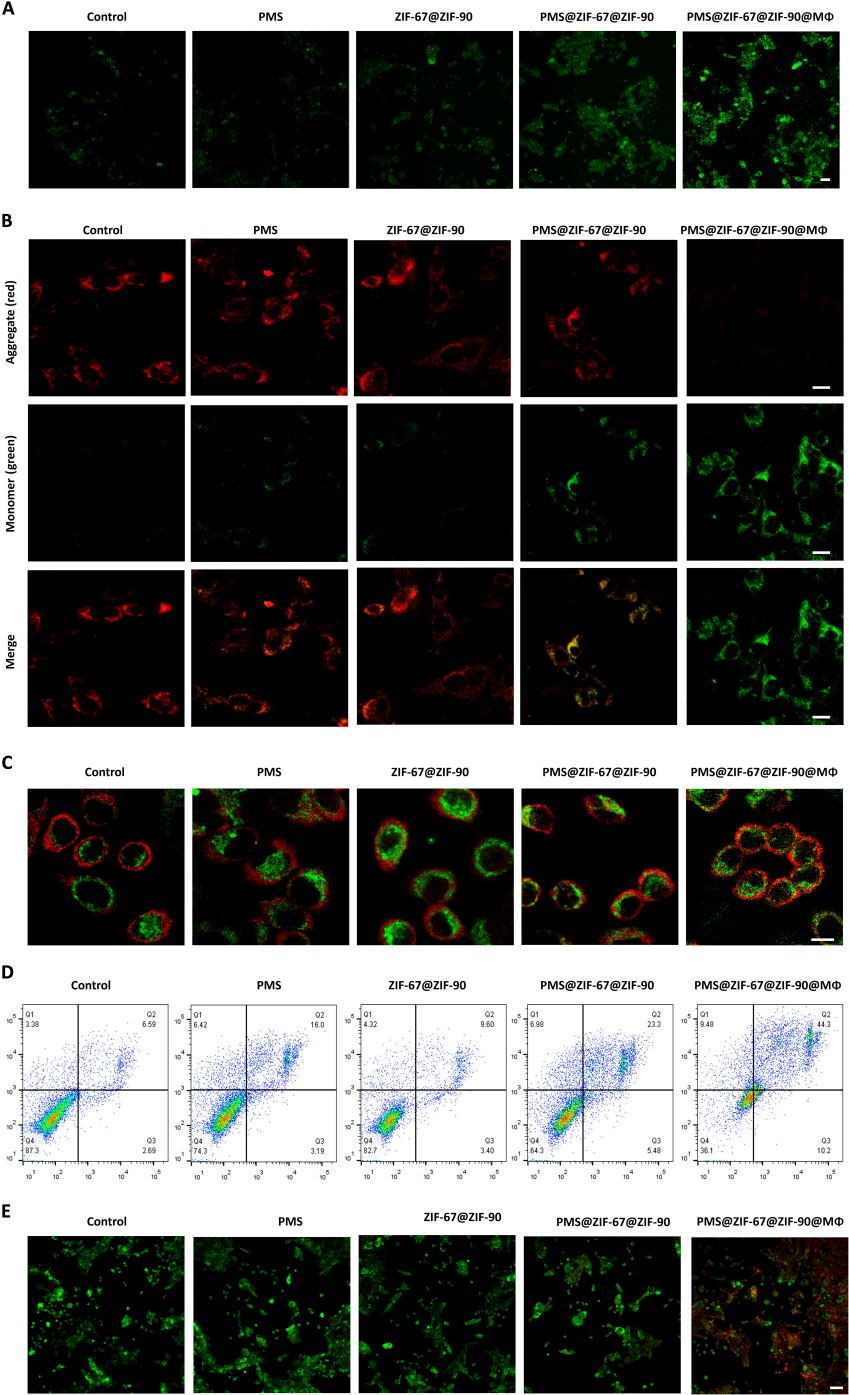
Evaluation of ROS production, mitochondrial apoptosis, and cell viability in LLC cells treated with various nanoformulations. (A) Detection of ROS production in LLC cytoplasm using DCFH-DA (2′,7′-dichlorofluorescein diacetate) with control, PMS, ZIF-67@ZIF-90, PMS@ZIF-67@ZIF-90, and PMS@ZIF-67@ZIF-90@Mφ. Green fluorescence indicates the presence of ROS (scale bar = 10 μm). (B) CLSM images of MMP by J-aggregate and J-monomer in LLC cells treated with different groups (scale bar = 10 μm). (C) CLSM images showing the expression of Cyp D in LLC cells treated with different groups. Green fluorescence represents mitochondria, while red fluorescence represents Cyp D expression (scale bar = 10 μm). (D) Flow cytometric analysis of apoptosis levels in LLC cells treated with different groups. (E) Live/dead cell images of LLC cells after treatment under different incubation conditions. Red fluorescence indicates dead cells, while green fluorescence represents living cells (scale bar = 50 μm).

The ROS generation capability of the macrophage missile was further examined in vitro. Fluorescence imaging using a hydroxyl radical detection kit showed that LLC cells treated with PMS@ZIF-67@ZIF-90@Mφ gave off strong green fluorescence, indicating efficient ·SO_4_^−^ generation (Fig. 5A) [39]. In comparison, there was a moderate increase in fluorescence intensity observed in the PMS and PMS@ZIF-67@ZIF-90 groups, which was attributed to the lack of a catalyst and efficient uptake by the cells, respectively. These results confirmed the essentiality of ATP-mediated ZIF-67 release for activating the catalytic efficacy. Having demonstrated the effect of ATP-regulated MOF nanoparticle catalyzed PMS to promote ·SO_4_^−^ production in mitochondria, the effects on mitochondrial impairment were also investigated. Mitochondrial membrane potential (MMP) is often used to characterize the accumulation of ROS in mitochondria [40,41]. The MMP changes were assayed in different groups-treated LLC cells stained by JC-1 dye as an indicator [42,43], which exists as a monomer at low MMP and produces green fluorescence, and gathers in the mitochondrial matrix at high MMP, forms the JC-1 aggregate, and generate red fluorescence, and thus the change in MMP can be analyzed by the shift in fluorescence. According to the CLSM shown in Fig. 5B, JC-1 in the PMS@ZIF-67@ZIF-90@Mφ group was predominantly present as monomers compared with other groups, revealing an abnormal MMP status and dramatic mitochondrial damage treated by this group. To further validate the mitochondrial abnormalities, the translocation of the cyclophilin D (Cyp D) protein from the cytoplasm to mitochondria was assessed, as it is associated with the formation of the mitochondrial permeability transition pore complex [44]. Mitochondria were labeled with MitoTracker@Green, and the translocation of Cyp D (red fluorescence) was assayed by immunostaining. A remarkable translocation of Cyp D molecules to the mitochondria was observed in the PMS@ZIF-67@ZIF-90@Mφ group (Fig. 5C), which had the highest colocalization rate among all the treatment groups, in agreement with the characterization results of MMP. Altogether, the findings clearly confirmed that PMS@ZIF-67@ZIF-90@Mφ induced the most vigorous mitochondrial damages, which was ascribed not only to the enhanced Mφ-assisted endocytosis but also to the mitochondrial targeting of ZIF-67@ZIF-90 and the subsequent oxidative stress generated by catalytic PMS.

The cytotoxic effects of macrophages missile were further evaluated on tumor cells. As shown in Fig. S7, no apparent therapeutic effect was observed in the PMS or ZIF-67@ZIF-90 groups, and PMS@ZIF-67@ZIF-90 had a certain extent of antitumor effect, suggesting that the catalytic ability of ZIF-67@ZIF-90 was activated by ATP-mediated degradative environment. With the Mφ targeting, PMS@ZIF-67@ZIF-90@Mφ demonstrated tremendous concentration-dependent cytotoxicity against LLC cells, implying that active endocytosis, mitochondrial targeting, and MOF-catalyzed ROS production can effectively induce cancer cells death, with only 28% of the cancer cells surviving within 24 h of incubation. The results were also validated by cell flow cytometry and live/death imaging, respectively (Fig. 5D and E), in which the PMS@ZIF-67@ZIF-90@Mφ group had the highest apoptosis rate of 44.3% and the most pronounced cell death. However, the cytotoxic effect of the PMS or MOFs alone on LLC cells was markedly reduced in comparison, with apoptosis rates as low as ~9.6%, and the cell proliferation reagent WST-1 assay and cell live/death imaging showed no obvious cell death. Together, these results emphasize the promising potential of PMS@ZIF-67@ZIF-90@Mφ in suppressing tumor cell proliferation, thus demonstrating its therapeutic advantage in tumor inhibition.

### Tumor targeting and antitumor effect of macrophages missile in vivo

**Fig. 6.**
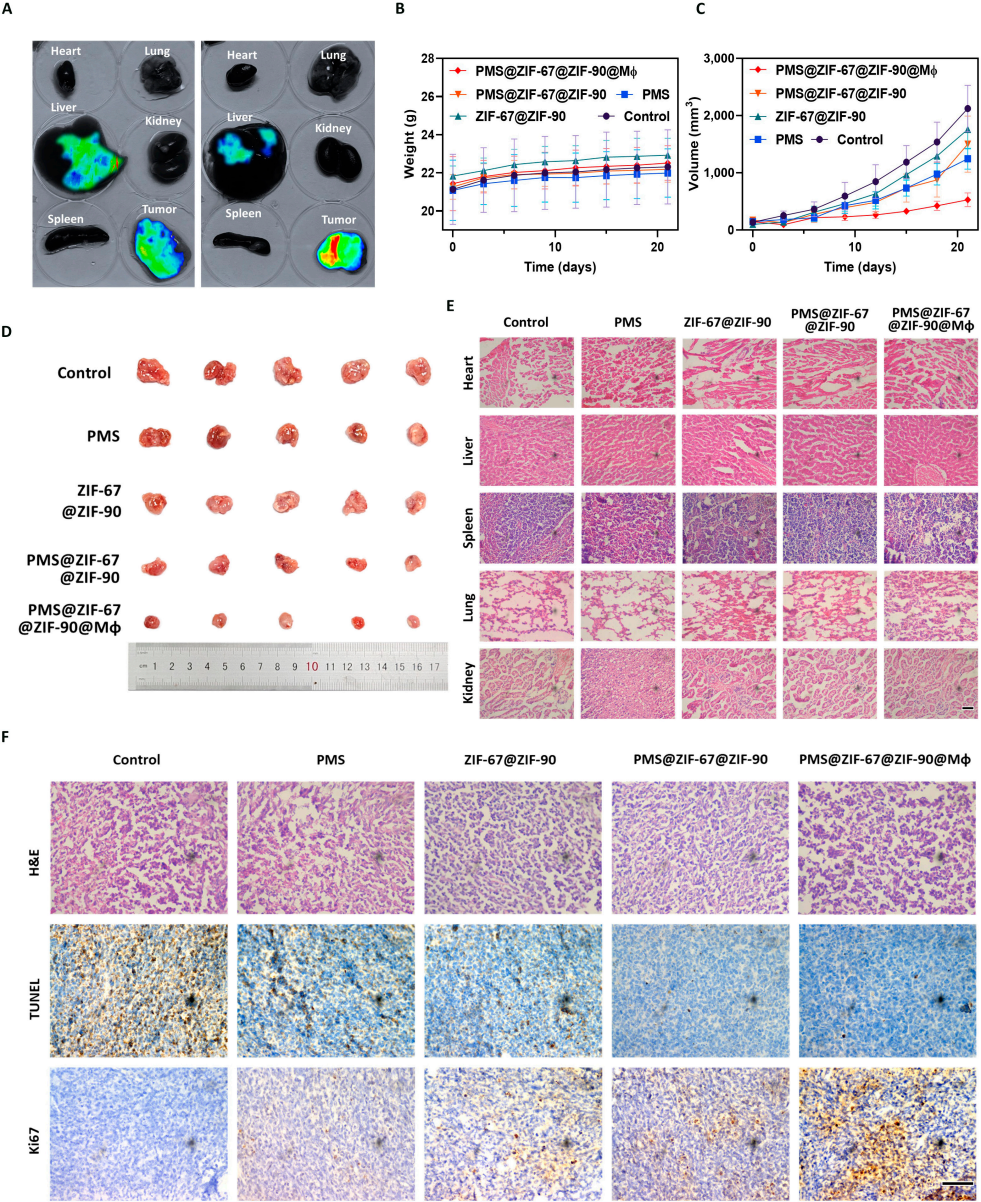
In vivo biodistribution of the macrophages missile, tumor growth inhibition, and histological analysis of tumor-bearing mice with different treatments. (A) The fluorescence images of the sacrificed mouse tissues, including heart, liver, spleen, lung, kidney, and tumor at 24 h after intravenous injection of PMS@ZIF-67@ZIF-90 and PMS@ZIF-67@ZIF-90@Mφ. (B) Tumor volume growth curves and (C) the body weight of mice among the different treatment groups. (D) Photographs of the stripped tumors from the sacrificed mouse on the 21st day after administration. (E) The morphology of sectioned organs from the nude mice model in the 5 groups via H&E staining (scale bar = 100 μm). (F) H&E, TUNEL, and Ki67 staining of sliced tumor tissues after different kinds of treatment (scale bar = 100 μm).

Following that, there was an in vivo fluorescence imaging experiments performed on LLC tumor-bearing mice to determine whether the macrophages missile is able to target the tumor tissue and to observe their biological distribution. This experiment involved the injection of ZIF-67@ZIF-90 and ZIF-67@ZIF-90@Mφ nanoparticles (labeled with Cy5.5) via the tail vein, and imaging was performed 24 h postinjection. The fluorescence signal was obviously stronger in the ZIF-67@ZIF-90@Mφ group compared to the ZIF-67@ZIF-90 group as shown in Fig. S8, which suggests that the nanoparticles accumulated in the tumor site, especially more pronounced at 24 h, which was attributed to the enhanced Mφ-mediated active targeting. In addition, when fluorescence imaging was performed on sections of tumors and organs in mice, it showed that the ZIF-67@ZIF-90@Mφ group had the highest fluorescence intensity in tumors, with reduced enrichment in organs such as the liver, where the fluorescence intensity was entirely lower than that of the ZIF-67@ZIF-90 group, and negligible accumulation in other normal organs (Fig. 6A). Such difference can be attributed to the rapid clearance by the immune system and the avoidance of nonspecific uptake [45]. These outcomes indicate that Mφ-based delivery system can also promote targeted accumulation of PMS@ZIF-67@ZIF-90 at tumor sites, enhancing the efficacy and specificity of PMS utilization, while reducing the toxicity of accumulation in other organs in vivo.

Based on the successful in vivo tumor targeting, the next step was to evaluate the in vivo antitumor effect of PMS@ZIF-67@ZIF-90@Mφ in LLC tumor-bearing mice. This animal test was initiated when the tumor size reached 50 mm^3^ (7 d posttumor cell inoculation). The mice were randomly divided into 5 groups and intravenously injected with PBS as control, PMS, ZIF-67@ZIF-90, PMS@ZIF-67@ZIF-90, and PMS@ZIF-67@ZIF-90@Mφ for every 3 d until day 21. The tumor growth and mouse body weight were also measured during the entire experiment. Tumor size recorded and the PMS@ZIF-67@ZIF-90 group showed minimal inhibitory of tumor growth, whereas the treatment with PMS alone resulted in a potent suppression of tumor growth (Fig. 6B), which was ascribed to the PMS-dependent ·SO_4_^−^ production [46]. In addition, there were no remarkable differences in the body weight of the mice in each treated group, indicating that the mice were well tolerated in the groups (Fig. 6C). The limited tumor inhibitory effect of PMS@ZIF-67@ZIF-90 alone is mainly attributed to its low accumulation and catalytic potency under environmental conditions. Also, the digital image of tumors after sacrifice was consistent with the result of volume curve, indicating that the targeted mitochondria damage has the best therapeutic outcome (Fig. 6D). In addition, the histological examinations of major organs, such as heart, liver, spleen, lung, and kidney, did not show any traces of treatment-induced damage in hematoxylin and eosin (H&E)-stained sections, which demonstrate the biocompatibility and biosafety of the macrophages missile strategy under clinically relevant conditions (Fig. 6E). As these results indicate, PMS@ZIF-67@ZIF-90@Mφ has strong antitumor effects while maintaining good biocompatibility and safety. To further evaluate the antitumor capacity of PMS@ZIF-67@ZIF-90@Mφ in vivo, immunohistochemical assays were performed on tumor tissues to detect the key markers associated with tumor growth (Fig. 6F). The apoptosis-related biomarker terminal deoxynucleotidyl transferase dUTP nick end labeling (TUNEL) signal was massively increased in PMS@ZIF-67@ZIF-90@Mφ-treated mice. The signal representing the proliferation-associated biomarker Ki67 was dramatically reduced in the PMS@ZIF-67@ZIF-90-treated group, especially in the PMS@ZIF-67@ZIF-90@Mφ-treated group. The above results suggested that PMS@ZIF-67@ZIF-90@Mφ can efficiently inhibit the tumor growth in vivo, while the oxygen-independent sulfate radicals were more effective in stimuli-responsive tumor nanotherapy.

## Discussion

The synthesis of core–shell MOF nanoparticles (ZIF-67@ZIF-90) and their subsequent functionalization to prepare a live Mφ-based delivery system represent a prominent advancement in targeted lung cancer therapy. The capability of these nanoparticles to catalyze PMS to generate sulfate radicals in an oxygen-independent manner is particularly noteworthy, as it circumvents the limitations associated with hypoxic tumor environments, which ensures more consistent and effective therapeutic outcomes. Furthermore, the innovative use of live Mφ as carriers through polyphenol-mediated interaction enhances the tumor-targeting capabilities of the drug-loaded nanoparticles. This approach not only improves the precision of drug delivery but also minimizes systemic toxicity, which is a critical concern in cancer therapy. The bioactivity preservation of the carrier cells and the extended clinical application window offer promising avenues for cancer treatment. The enhanced tumor penetration and potent antitumor effects observed in this study highlight the potential of this system to completely improve patient outcomes. This work emphasizes the crucial role of nanotechnology in developing sophisticated drug delivery systems that can overcome traditional therapeutic challenges.

Despite the promising results, there are several limitations to this study. The long-term stability and potential immunogenicity of the ZIF-67@ZIF-90 nanoparticles need further investigation. Understanding how these nanoparticles interact with the immune system over extended periods is essential to ensure their safety and efficacy. Additionally, the exact mechanisms underlying the minimized endocytosis by Mφ and the retention of their biological activity need to be elucidated further. The completed studies should be conducted to assess the pharmacokinetics, biodistribution, and long-term effects of the nanoparticles. It is also crucial to explore the scalability of the synthesis process to ensure that the nanoparticles can be produced consistently and in sufficient quantities for clinical use. Furthermore, the development of standardized protocols for the functionalization and attachment of nanoparticles to Mφ will be vital to ensure reproducibility and reliability across different research settings.

Overall, this study lays a solid fundament for the development of advanced, targeted cancer therapies and highlights the transformative potential of combining nanotechnology with biological systems. By addressing the outlined limitations and taking the necessary steps toward clinical translation, this innovative approach has the potential to thoroughly improve cancer treatment outcomes.

## Materials and Methods

### Materials

Co(NO_3_)_2_·6H_2_O, Zn(NO_3_)_2_·6H_2_O, methanol, 2-methylimidazole (98%), 2-ICA (98%), PMS(KHSO_5_·0.5KHSO_4_·0.5K_2_SO_4_), TA (98%), and TBA were purchased from Sigma-Aldrich. All products were used without further purification.

### Synthesis of nanosized ZIF-67

Co(NO_3_)_2_·6H_2_O (0.59 g) and polyvinyl pyrrolidone (0.5 g) were dissolved in 50 ml of methanol. 2-Methylimidazole (0.66 g) and triethylamine (40 μl) were dissolved in another 50 ml of methanol. Then, 2-methylimidazole solution was poured into Co(NO_3_)_2_·6H_2_O solution under magnetic stirring for 10 min and aged for 24 h at room temperature. The product was collected by centrifugation and washed with methanol 3 times.

### Synthesis of core–shell ZIF-67@ZIF-90 and PMS@ ZIF-67@ZIF-90 nanoparticles

The prepared ZIF-67 (10 mg) and Zn(NO_3_)_2_·6H_2_O (3.7 mg) were dispersed in 6.25 ml of ethanol/water (1:1, v/v) solution, followed by addition to 6.25 ml of ethanol/water (1,1, v/v) solution dissolved with 2-ICA (4.8 mg) and stirred at room temperature for 3 h. The product (ZIF-67@ZIF-90) was centrifuged and washed 3 times with methanol. By mixing PMS with ZIF-67@ZIF-90 solution (1,1, w/w) for 60 min, PMS@ ZIF-67@ZIF-90 was obtained after centrifugation and washing 3 times with H_2_O.

### Synthesis of macrophages missile nanoparticles

Macrophages (5 × 10^7^) were suspended in 500 μl of PBS (pH = 5) solution containing 400 μg·ml^−1^ ZIF-67@ZIF-90 nanoparticle. After vortexing for 10 s and incubating for 30 s, 500 μl of 40 μg·ml^−1^ TA in PBS (pH = 5) solution was added with vigorous stirring for 30 s. The formed macrophages missile was then rinsed with PBS (pH = 7.4) and stored in PBS (pH = 7.4).

### In vitro RB degradation evaluation

Tube I: RB aqueous solution as control, Tube II: Tube I with 150 μg·ml^−1^ PMS, Tube III: Tube II with 10 μg·ml^−1^ ZIF-67, Tube IV: Tube III with quencher tetra-butanol (TBA), Tube V: Tube III with quencher ethanol, Tube VI: Tube II with 10 μg·ml^−1^ ZIF-67@ZIF-90, Tube VII: Tube VI with 1.0 mM ATP. TBA was leveraged as a quencher for ·OH, while ethanol was utilized as a quencher for ·SO_4_^−^ and ·OH. The absorption was detected after 60 min.

### Intracellular ROS production

LLC cells were cultured overnight in 6-well plates (1 × 10^5^ cells per well), then coincubated with PMS, ZIF-67@ZIF-90, PMS@ZIF-67@ZIF-90, and PMS@ZIF-67@ZIF-90@Mφ for 24 h. DCFH-DA was added to the above dishes and coincubated for 30 min. Finally, the intracellular fluorescence intensity of DCF was observed by CLSM to evaluate the production of ROS.

### In vitro cytotoxicity

LLC cells were inoculated into 96-well culture plates (5 × 10^3^ cells per well) overnight, followed by replacing the original medium with 100 ml of fresh culture medium containing different concentrations of PMS, ZIF-67@ZIF-90, PMS@ZIF-67@ZIF-90, and PMS@ZIF-67@ZIF-90@Mφ. After 24 h of incubation, the cell proliferation reagent WST-1 assay was used to assess the viability of cells (*n* = 6).

### In vitro antitumor performance

LLC cells were coincubated with PMS, ZIF-67@ZIF-90, PMS@ZIF-67@ZIF-90, and PMS@ZIF-67@ZIF-90@Mφ after they adhered to the wall of the dish overnight. After another 18 h of incubation, the cells were trypsinized and washed with PBS. Then, the Annexin V (5 μl) and propidium iodide (5 μl) were added to distinguish cells in different apoptotic states. Finally, the apoptosis condition of different groups was determined by flow cytometry. LLC cells were planted into confocal dishes with a density of 1 × 10^5^ cells per well and incubated for 24 h to proceed live-dead cell staining assay. Medium containing PMS, ZIF-67@ZIF-90, PMS@ZIF-67@ZIF-90, and PMS@ZIF-67@ZIF-90@Mφ was used to replace the original medium. After another 18 h of incubation, the cells were washed with PBS and stained by 100 μl of the solution of Calcein-AM (1 μM) and propidium iodide (5 μM) for 15 min. The fluorescence intensity was observed by CLSM.

### Animal experiments

BALB/c mice, specifically male mice at the age of 6 to 8 weeks, were obtained from Guangdong Medical University. All experimental procedures involving animals were approved by Guangdong Medical University and used in compliance with a local ethics committee (Permit Number: AHGDMU-LAC-B-202312-0091).

### In vivo antitumor study

LLC cells (1 × 10^6^) were injected subcutaneously into the BALB/c nude mice (5 wk old) to build the tumor models, and when the volume reached around 50 mm^3^, the mice were divided into 5 groups (*n* = 6). The mice in each group were assigned to receive different treatments, including control, PMS, ZIF-67@ZIF-90, PMS@ZIF-67@ZIF-90, and PMS@ZIF-67@ZIF-90@Mφ. The treatment administration involved intravenous injection through the tail vein on the first day, followed by subsequent administrations every 3 d. The tumor volume and body weight of mice were measured every 3 d. The tumor volume was calculated using the formula *V*_tumor_ = *LW*^2^/2 (*L*: maximum diameter of tumor, *W*: minimum diameter of tumor). Twenty-one days later, the mice were euthanized, and the tumors were collected for further staining and analysis.

### Statistical analysis

Quantitative data were expressed as the mean ± SD. Two-way analysis of variance was employed with Tukey’s post hoc test, and differences were regarded significant if *P* < 0.05 (*P < 0.05, **P < 0.01, ***P < 0.001). All statistical analyses were performed with GraphPad Prism (8.0).

## Data Availability

The data that support the findings of this study are available from the corresponding authors upon reasonable request.
