# Supplementary material for: Utilizing Macrophages Missile for Sulfate-Based Nanomedicine Delivery in Lung Cancer Therapy
Source: Research (Wash D C). 2024 Aug 14;7:0448. doi: 10.34133/research.0448 (PMC11301451; doi:10.34133/research.0448)
Supplement: Supplementary 1 — Figs. S1 to S8 [file research.0448.f1.docx]

Supplementary Materials

Fig. S1. Characterization of size and Zeta potential of nanoparticles.

Fig. S2. SEM analysis of synthesized different nanoparticles.

Fig. S3. UV-Vis characterization of synthesized different nanomaterials.

Fig. S4. FT-IR characterization of synthesized different nanomaterials.

Fig. S5. TEM characterization of sectioned macrophages missiles.

Fig. S6. Assessment of cellular uptake and lysosomal co-localization of nanomaterials in LLC Cells.

Fig. S7. Assessment of cytotoxicity of nanomaterials against LLC cells.

Fig. S8. Assessment of nanomaterials targeting ability by in vivo fluorescence imaging.


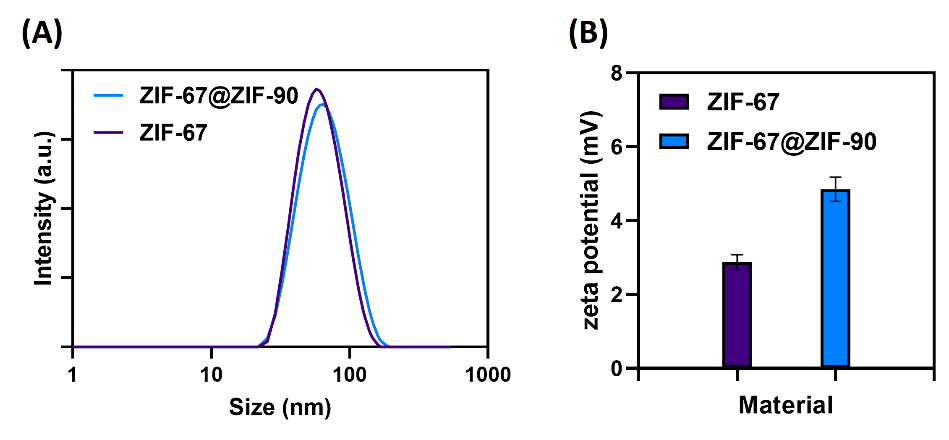


***Fig. S1. Characterization of size and Zeta potential of nanoparticles.*** *Hydrodynamic diameter distribution (A) and Zeta potential (B) of ZIF-67 and ZIF-67@ZIF-90 in aqueous solution.*


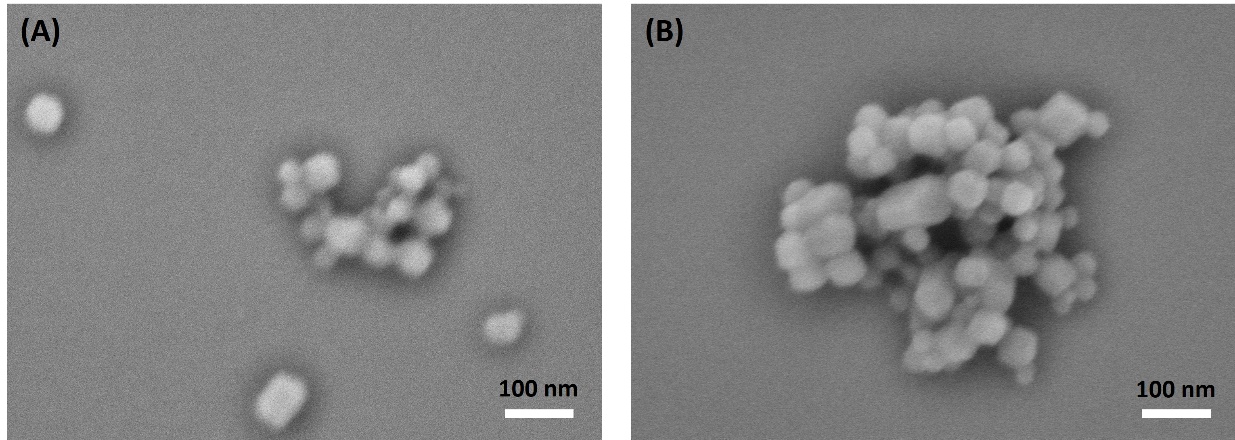


***Fig. S2. SEM analysis of synthesized different nanoparticles.*** *SEM images of ZIF-67 (A) and ZIF-67@ZIF-90 (B).*


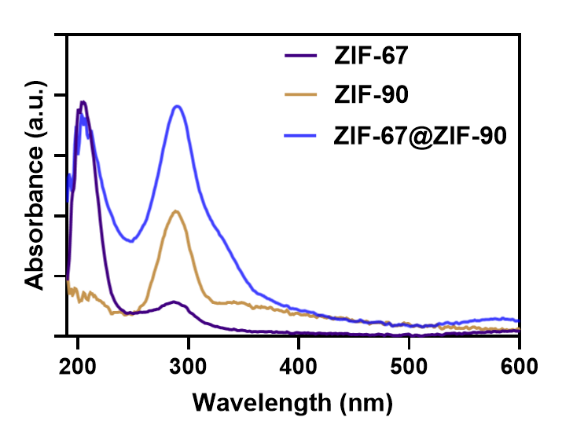


***Fig. S3. UV-Vis characterization of synthesized different nanomaterials.*** *UV-Vis absorption spectra of ZIF-67, ZIF-90, and ZIF-67@ZIF-90 nanoparticles.*


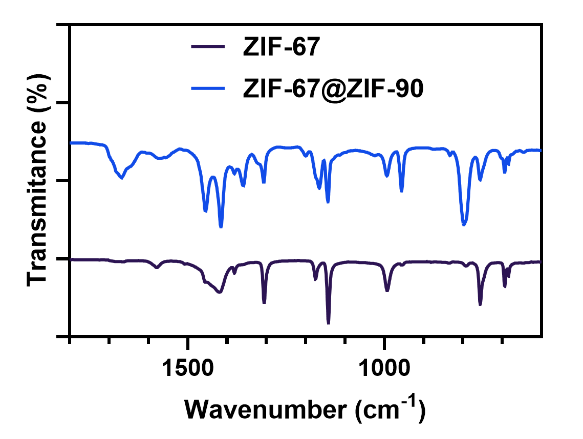


***Fig. S4. FT-IR characterization of synthesized different nanomaterials.*** *FT-IR spectra of ZIF-67 and ZIF-67@ZIF-90 nanoparticles.*


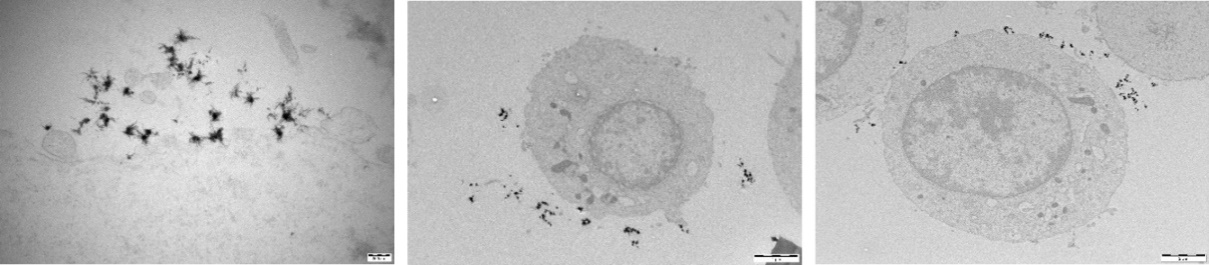


***Fig. S5. TEM characterization of sliced Macrophages Missiles.*** *TEM images of the sectioned ZIF-67@ZIF-90@Mφ, the ZIF-67@ZIF-90 nanoparticle attached on the Mφ surface.*

**
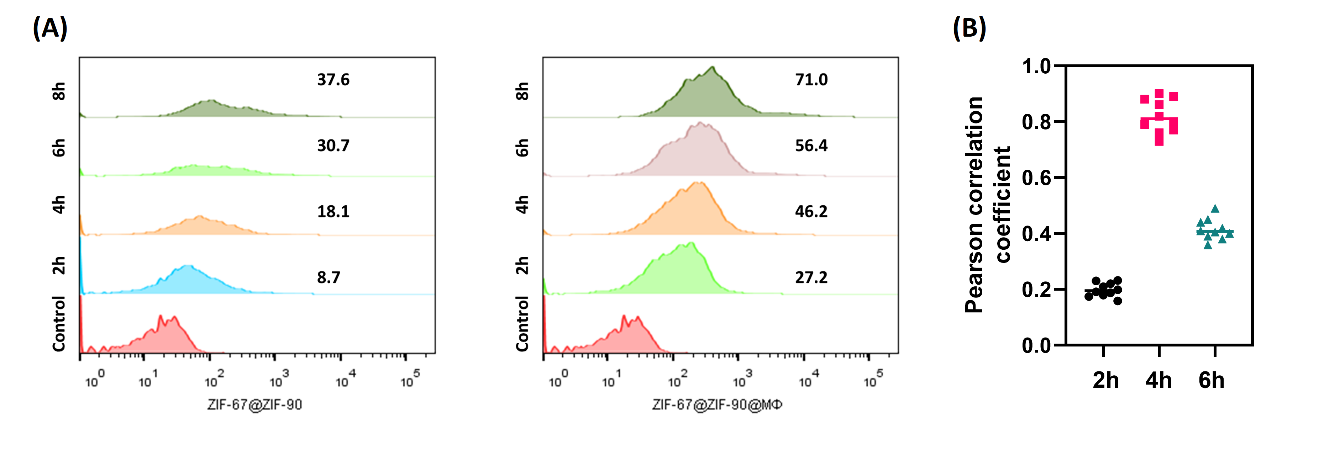
**

***Fig. S6.*** ***Assessment of cellular uptake and lysosomal co-localization of nanomaterials in LLC Cells.*** *(A) Flow cytometry analysis of LLC cells after incubated with ZIF-67@ZIF-90 and ZIF-67@ZIF-90@Mφ, using LLC without any treatment as a blank group. (B) The quantitative analysis of co-localization of ZIF-67@ZIF-90 with lysosomes labeled with LysoTracker@Green. The Pearson correlation coefficients are close to 1 if they are highly co-localized (n = 10).*


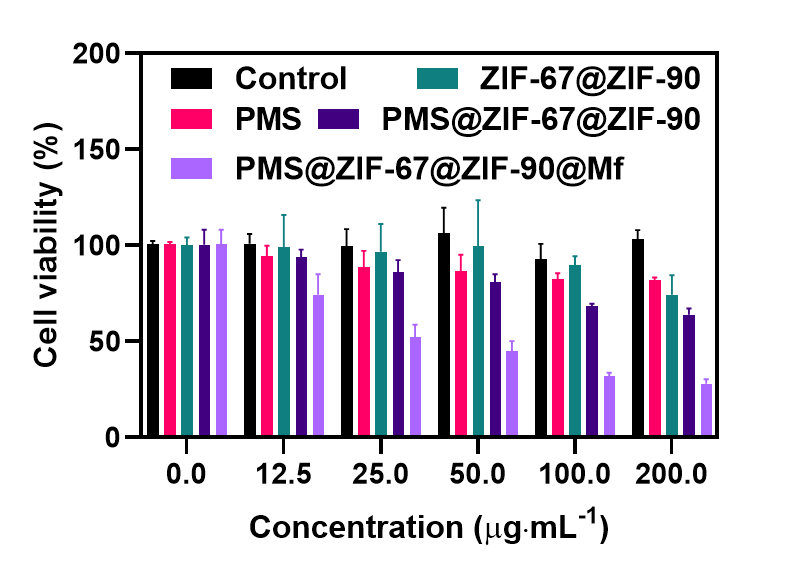


***Fig. S7. Assessment of cytotoxicity of nanomaterials against LLC cells.*** *The cytotoxicity of Control, PMS, ZIF-67@ZIF-90, PMS@ZIF-67@ZIF-90 and PMS@ZIF-67@ZIF-90@Mɸ against LLC cells were measured by WST-1 assay, respectively.*


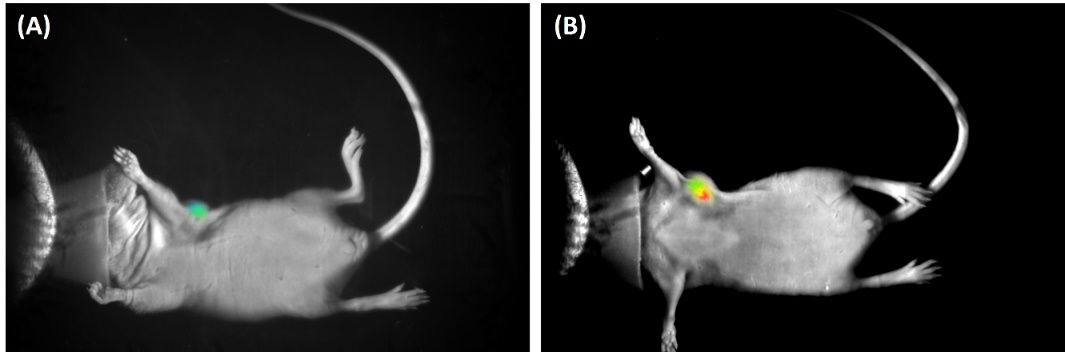


***Fig. S8. Assessment of nanomaterials targeting ability by in vivo fluorescence imaging.*** *In vivo fluorescence images of LLC tumor-bearing mice after intravenous injection of Cy5.5 labeled ZIF-67@ZIF-90 (A) and ZIF-67@ZIF-90@Mφ (B), respectively.*
